# Plotting a future for Amazonian *canga* vegetation in a *campo rupestre* context

**DOI:** 10.1371/journal.pone.0219753

**Published:** 2019-08-05

**Authors:** Daniela C. Zappi, Marcelo F. Moro, Barnaby Walker, Thomas Meagher, Pedro L. Viana, Nara F. O. Mota, Mauricio T. C. Watanabe, Eimear Nic Lughadha

**Affiliations:** 1 Instituto Tecnológico Vale, Belém, Pará, Brazil; 2 Museu Paraense Emílio Goeldi, Coordenação Botânica, Belém, Pará, Brazil; 3 Instituto de Ciências do Mar (Labomar), Universidade Federal do Ceará, Fortaleza, Ceará, Brazil; 4 Conservation Science Department, Royal Botanic Gardens, Kew, Richmond, Surrey, United Kingdom; 5 School of Biology, University of St Andrews, St Andrews, Fife, United Kingdom; University of Florida, UNITED STATES

## Abstract

In order to establish effective conservation strategy, drivers of local and regional patterns of biodiversity need to be understood. The composition of local biodiversity is dependent on a number of factors including evolution and redistribution of lineages through dispersal and environmental heterogeneity. Brazilian *canga* is characterised by a ferrugineous substrate, found both in the Iron Quadrangle of Minas Gerais and in the Carajás mountains in Amazonia. *Canga* is one of several specialised habitat types comprising Brazilian *campo rupestre*, a montane vegetation found within or adjacent to several major Brazilian bioregions, including the Atlantic Forest and Amazonia, with exceptionally high levels of diversity and endemism arising from both history of dispersal and environmental variation. In order to inform biodiversity conservation for *canga*, and more broadly for *campo rupestre*, we performed floristic and phylogenetic analyses investigating affinities between 28 sites on different substrates (*canga* and quartzite) and geographic locations (Carajás, Pará [Amazonia]; Cadeia do Espinhaço, Minas Gerais; Chapada Diamantina, Bahia). Through analysis of 11204 occurrences of 4705 species of angiosperms, we found that Amazonian Carajás *canga* plant communities formed a cohesive group, distinct from species assemblages found in Eastern Brazil (Minas Gerais, Bahia), either on *canga* or quartzite. The phylogenetic megatree of species across all sites investigated shows associations between certain clades and Amazonian *canga*, with few shared species between the Amazonian Carajás and Eastern Brazil sites, while the floristic comparison shows high levels of heterogeneity between sites. The need for reserves for Amazonian Carajás *canga* has been recognized and addressed by the creation of a national park. However, current sampling does not provide sufficient reassurance that the *canga* areas now benefitting from full legal protection adequately represent the regional *canga* flora.

## Introduction

The composition of biodiversity in any given locality is the result of historical and ongoing plant dispersal mediated by local environmental conditions. It is now well established that patterns of biodiversity are strongly influenced by substrate and microclimate [1–3]. Conservation strategy needs to take into account such drivers of local biodiversity in order to be effective at larger scales.

Brazil is home to major biogeographical regions of international importance in terms of biodiversity and ecosystem services, and notable among these is Amazonia, that comprises roughly 40% of Brazil. A number of recent studies centred on Amazonia have focussed on plant taxa with dispersal patterns likely to be based on past human influence [4,5], long distance dispersal [6] or a combination of both [7], and have suggested that Amazonia is a single coherent biome. In reality, there is considerable environmental heterogeneity across this region that has a substantial impact on local biodiversity, and thus has significant implications for conservation. In addition to conservation strategy, understanding of impacts of environmental drivers of biodiversity also has potential to inform understanding of ecosystem services, such as carbon turnover and storage, at larger regional scales [8,9].

The Brazilian *campo rupestre* is a complex mosaic dominated by open vegetation including meadows, scrubland, grasslands and open savannas, as well as forest groves (known as *capões*) and gallery forests [3,10]. *Campo rupestre* occupies montane areas within major Brazilian biomes, including Amazonia, and is extremely high in plant diversity and endemism, at least in Eastern Brazil [11]. The best studied sites of *campo rupestre* are found in the highlands of Eastern Brazil, spanning the states of Minas Gerais (Cadeia do Espinhaço) and Bahia (Chapada Diamantina) [10,12–14]. Earlier studies highlight floristic differences between *campo rupestre* vegetation growing on quartzite and iron-rich (*canga*) formations [3,15] but conclude that both of these systems can be recognised as old, climatically-buffered infertile landscapes, termed Ocbils [16,17].

The Serra dos Carajás, located within Amazonia (State of Pará), is home to one of the largest mineral provinces in the world [18], with mountain tops covered with grasslands on *canga* surrounded by lowland rainforest [19]. The magnitude of the iron-ore reserves found in Amazonian *canga* puts considerable conservation pressure on *campo rupestre* on *canga* [19]. The ‘Iron Quadrangle’ of Minas Gerais and the Serra dos Carajás (Pará) encompasses two of the largest open-cast iron mining areas in the world, extracting billions of dollars’ worth of mineral per year, making Brazil second only to Australia in terms of iron-ore exports [20,21].

While the relatively accessible *campo rupestre* of Eastern Brazil has received ongoing botanical attention over the last four decades, until very recently there was no comprehensive treatment of the floristic composition of the *campo rupestre* in Amazonian Carajás, despite the start of large-scale iron extraction at the turn of last century [22]. Until 2014, known plant species of *campo rupestre* of Carajás were estimated at 250 species [23]. However, from 2015 to 2018, the *Flora of Carajás* project [19,22,24] made unprecedented efforts to collect, compile, and document the flora of several *canga* mountaintops, both in the *Carajás National Forest*, a sustainable exploitation conservation area (IUCN conservation category VI [25]), and in the recently created *Parque Nacional dos Campos Ferruginosos*, a national park (IUCN category II [25]). The resulting *Flora of Carajás* [19] is the first authoritative comprehensive list for Carajás and documents more than 1000 land plant species, of which 855 are Angiosperms distributed in 115 plant families that occur on *canga*. Four genera and 38 species (Giulietti et al. under review) are reported as endemic to the Carajás *canga* outcrops, that add up to 120 km^2^ in total area [19].

We used the *Flora of Carajás* dataset to analyse the biogeography of *campo rupestre* on Amazonian Carajás *canga* and compare it to the *campo rupestre* of Eastern Brazilian highlands from Minas Gerais and Bahia. We aimed to investigate the similarities of *campo rupestre* flora and to discuss conservation implications for areas currently impacted by mining. In this first ever biogeographical study of Amazonian Carajás *canga*, we addressed the following questions. First, does Amazonian Carajás *canga* present floristic affinities with vegetation on *canga* in the highlands of Eastern Brazil, or does their flora comprise two very distinct groups, differing in species composition? Second, if *campo rupestre* in Eastern Brazil and *canga* vegetation in the Amazon form distinct floristic groups, can we identify lineages more associated with *canga* in Eastern Brazil and *canga* in Amazonia? Finally, can quantification of floristic differences between Amazonian Carajás *canga* sites inform future conservation management strategy?

## Material and methods

### Site survey collation

**Fig 1:**
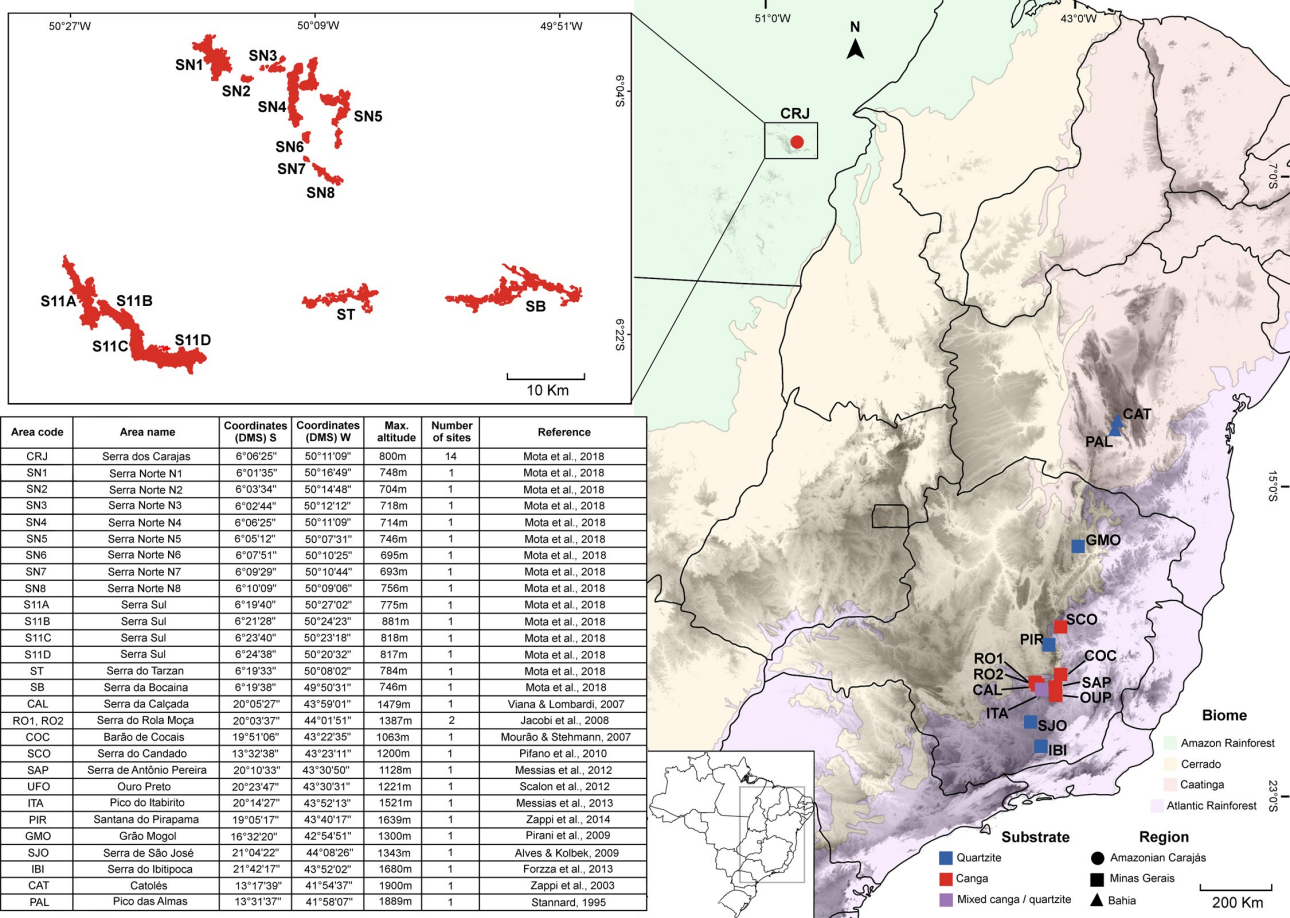
Map of study sites in Eastern Brazil and Amazonian Carajás (CRJ) showing canga (red) and quartzite (blue) sites. Carajás sites are magnified to show Serra Norte (SN1-8), Serra Sul (S11A-D), Serra do Tarzan (ST) and Serra da Bocaina (SB). Brazilian Biomes are Amazon Rainforest (pale green), Cerrado (pale yellow), Caatinga (pale orange) and Atlantic Rainforest (lilac).

**Fig 2:**
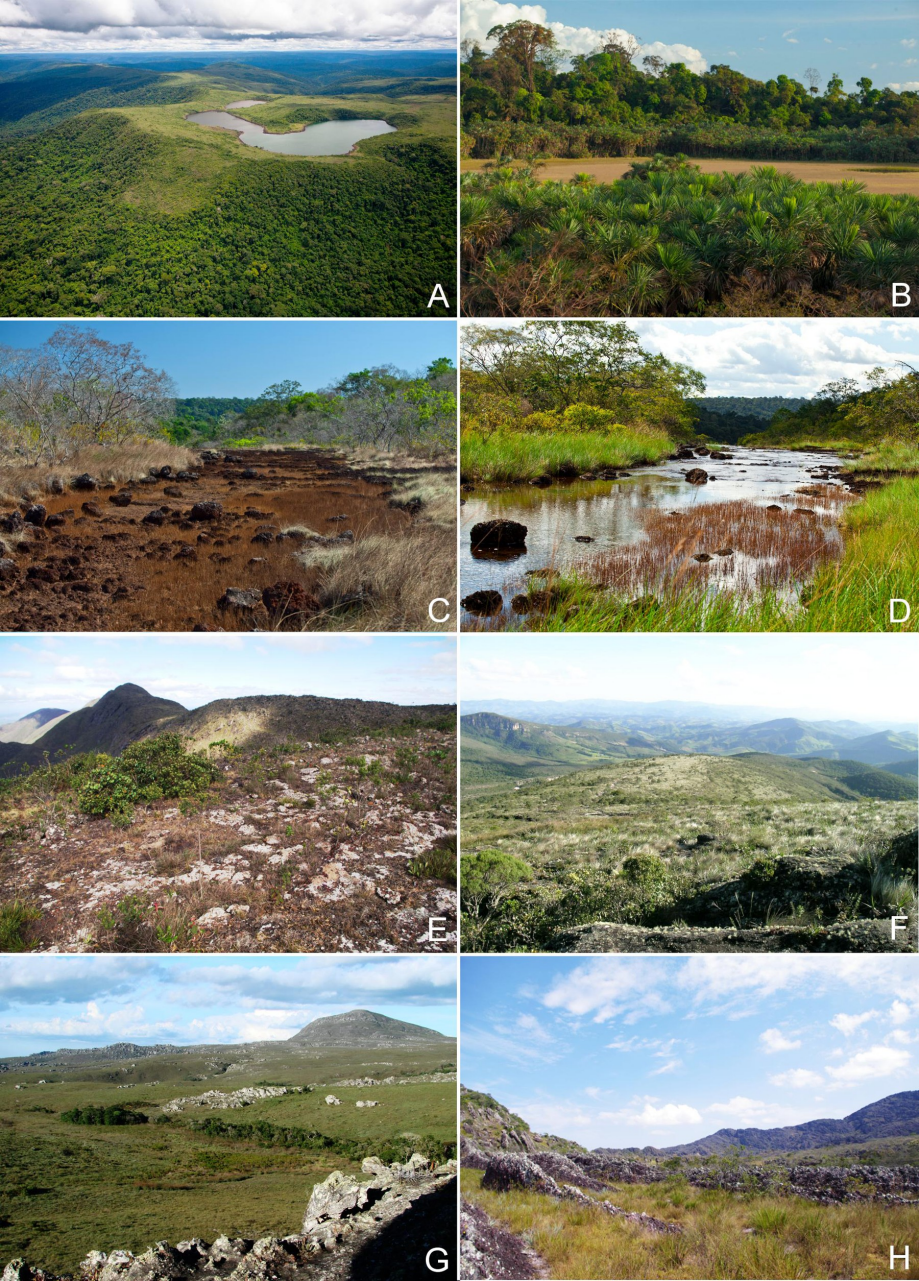
A-D: Amazonian *canga* in Pará, Brazil; A. Aerial view of the Lagoa das Três Irmãs, Serra Sul, FLONA Carajás showing dense forest reaching the edge of the open vegetation; B. Temporary lagoon at the Serra da Bocaina, Parque Nacional dos Campos Ferruginosos; C-D. Serra Sul landscape during the dry (C) and rainy season (D). E. Eastern Brazil *canga* at the Serra do Capanema, Minas Gerais. F-H: Eastern Brazilian *campo rupestre* on quartzitic substrate; F. Serra do Ibitipoca, Minas Gerais; G. Diamantina plateau in Minas Gerais; H. Serra do Barbado, near Catolés, Chapada Diamantina, Bahia. (Photos A-D João Marcos Rosa, E-H PLV).

**Fig 3:**
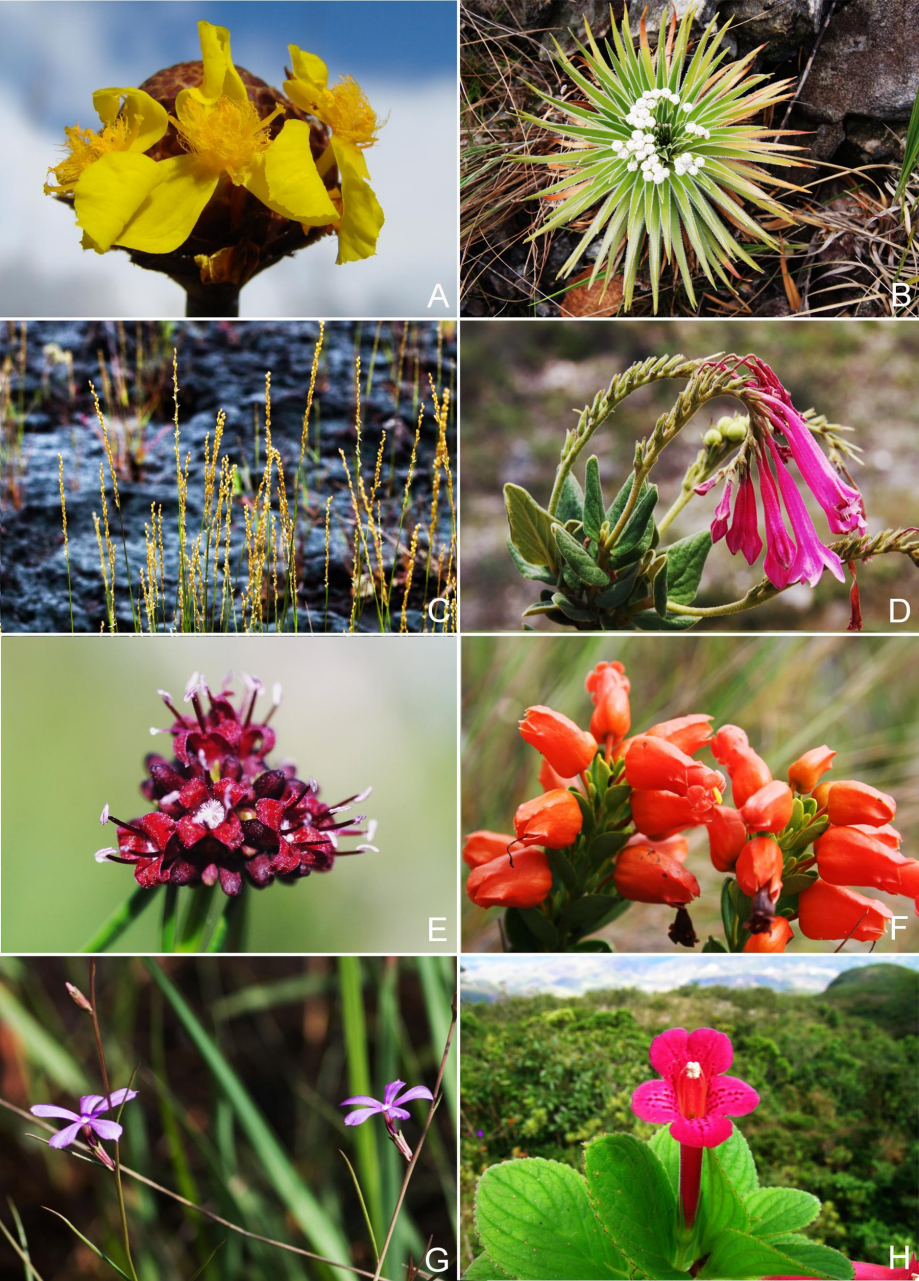
A. Critically Endangered *Xyris platystachya* (Xyridaceae) from the Serra do Cipó, Minas Gerais; B. *Paepalanthus eriophaeus* (Eriocaulaceae) collected at the Serra do Cipó, Minas Gerais; C. *Sporobolus multiramosus* (Poaceae), a rare species from the Amazonian *canga* in Carajás, Pará; D. *Spigelia sellowiana* (Loganiaceae) in Minas Gerais; E. *Borreria elaiosulcata* (Rubiaceae), restricted to the Amazonian *canga* in Carajás, Pará; F. Endangered *Physocalyx scaberrimus* (Orobanchaceae) from the Serra do Cipó, Minas Gerais; G. *Buchnera carajasensis* (Orobanchaceae), endemic to the Amazonian *canga* in and around Carajás, Pará; H. *Vanhouttea hilariana* (Gesneriaceae), from the southernmost quartzitic *campo rupestre* of Minas Gerais. (Photos A, C, E, G, H PLV, B DCZ, D William Milliken, F Leandro Freitas).

To perform the biogeographical analyses of the flora of *campo rupestre* we built a database showing the presence of species on a number of sites across the geographical distribution of this habitat. We used as starting point the floristic database of *campo rupestre* in eastern Brazil (Espinhaço, in Minas Gerais, and Chapada Diamantina, in Bahia) prepared by Zappi et al. [3]. To this database we added the survey data on the floristic composition of vegetation on *canga* in Carajás [19]. During the *Flora of Carajás* project [19,22,24] we recorded the occurrence of species in individual sites, under “Field permit number ICMBio 63324–1". This resulted in species lists for 14 sites in Eastern Brazil highlands: 12 in the Cadeia do Espinhaço of Minas Gerais (hereafter Minas Gerais) and 2 in the Chapada Diamantina of Bahia (hereafter Bahia) on *canga* and/or quartzite substrates (Fig 1). Images to illustrate the different landscapes (Fig 2) and characteristic plant species (Fig 3) are provided.

Fieldwork for the *Flora of Carajás* project [19,22,24] recorded floristic data for 14 individual mountaintop sites across four locations: Serra Norte (8 sites) and Serra Sul (4 sites), all in the Carajás National Forest (hereafter FLONA Carajás), and Serra da Bocaina and Serra do Tarzan, which are physically part of the Serra Sul but are now included in the Campos Ferruginosos National Park (PNCF). The FLONA Carajás is a national protected area that allows sustainable iron-ore mining (IUCN category VI [25]). In contrast, the PNCF is a category II [25] protected area, where it is expected that the *canga* will be completely conserved. All Angiosperm records were stored in a database using the ´plotsamples´ module of Brahms software [26]. Names qualified with *´aff*.*´* and ´*sp*.*´* in the original lists (e.g. *Eriocaulon* aff. *setaceum*, *Ruellia* sp.1) were removed from the study because their identity was not fully ascertained. Species deemed exotic invasive following definitions by [27] and [28] complemented by [29] were also excluded to avoid portraying inaccurate relationships between sites sharing alien species.

Plant nomenclature followed the Brazilian List of Plants and Fungi [30] through a species list extracted from Brahms and imported into the “PlantMiner” (www.plantminer.com) script [31] and “flora” package for R environment, which correct plant names following the Flora of Brazil database (BFG 2015). Infra-specific categories (subspecies, varieties and forms) were treated at specific level.

Each site in our database was classified according to its geographic location (Amazonian Carajás, Minas Gerais, and Bahia) and substrate (*canga* or quartzite). There was one Minas Gerais site that included both *canga* and quartzite that was designated as ´mixed´ [32].

### Multivariate analyses

From the database showing the species list for each of our 28 sites (14 in Amazonian Carajás, see Supporting information—S1 Table, 12 in Minas Gerais, 2 in Bahia) we created a presence-absence matrix (see Supporting information—S2 Table), showing the occurrence of each species in each site, from which we calculated the beta-diversity between all pairs of sites using Bray-Curtis (Sorensen) distances [33]. The beta-diversity matrix with the ecological distance between all sites was analysed using non-metric multidimensional scaling (NMDS) and UPGMA grouping analysis using “Vegan” and “Stats” packages for R [34,35].

To test whether the floristic groups defined by geographical region and substrate were significantly different, we used the script ANOSIM (“analysis of similarities”) implemented in Vegan R package [34].

After these subcontinent-wide analyses, we focused in more detail on the biogeography of the mountaintops of Amazonian Carajás, aiming to quantify heterogeneity and biogeographical relationships of the flora among different mountaintops, using Bray-Curtis distances for NMDS and UPGMA analyses.

### Phylogenetic reconstruction and tree visualization

To evaluate the phylogenetic structure of *campo rupestre* assemblages in each geographical region and substrate (*canga* or quartzite) we created a phylogenetic tree showing the evolutionary relationship among all species in our database. We used the megatree R20160415.new [36], reflecting phylogenetic relationship between plant families as recognized by APG [37], with clade node dates estimated by Magallón et al [38]. We loaded our species list, R20160415, and ages_magallon_PL age file into Phylocom 4.1 [39] to create our own dated megatree showing phylogenetic relationships among all species recorded in our database for the *campo rupestre* (see Supporting information—S1 Text and S4 Fig). We used iTOL [40] to visualize our *campo rupestre* megatree, indicating the occurrence of each species by geographic region and substrate, highlighting selected plant families.

### Differential representation of clades across areas (nodiv)

We used the *nodiv* package in R [41] to identify clades that exhibited significant differences in distribution between sites. This package implements a node-based analysis of species representation based around two metrics: a specific overrepresentation score (SOS) for each node in the phylogenetic tree at each site, and a geographical node divergence (GND) for each node in the phylogenetic tree. The SOS is calculated as the difference in standardised residual of species richness between the two sister clades descending from each node. The species richness of each sister clade is standardised using the mean and standard deviation of a sampled distribution of species richness for each clade, which is generated by randomising the species presence matrix. These sampled distributions of species richness are also used to calculate a statistical p-value for significant over- or under-representation of a specific clade. The GND for a particular node is then calculated as the mean log-odds of the p-value for that node at every site. Thus, GND allows one to determine whether an individual sister clade descendent from a given node is more represented in a group of sites, while the other sister clade of the same node is more represented in a different group of sites.

We identified clades with significant divergence in representation between sites as those with a GND greater than 0.65, following [41]. We then examined the difference in representation of the child clades of those divergent clades by comparing the SOS score at each site.

### Estimating species richness for Amazonian Carajás

Although only c. 250 species were previously recorded from the *canga* of Amazonian Carajás [22,23] before the *Flora of Carajás* project [19,22,24], the recently concluded flora lists 830 native Angiosperm species. To estimate how many species are expected to occur in this habitat, including species as yet unrecorded, we used two methods: extrapolation of the sampling curves and estimation of asymptotic (total) richness. For our Carajás inventory of 14 mountaintops, each plateau was considered a sampling site (S1 Table). We then rarefied (interpolated) the sampling to see whether the curve of sampled richness was stabilising, and estimated total, asymptotic richness with ICE, Chao 2, Jacknife 1 and Jacknife 2 estimators [42,43]. We also extrapolated our sampling effort to 28 mountaintops (twice our actual sampling) to evaluate whether or not a considerable increase in species richness is expected with more sampling in the region. Both methods aimed at estimating the richness of angiosperm flora of the Amazonian Carajás *canga* and verifying whether current knowledge is comprehensive. We performed rarefaction, richness estimation and extrapolation using EstimateS 9.1 software [44]. A methodology flowchart is provided (see Supporting information—S3 Fig).

## Results

### Overview

**Fig 4:**
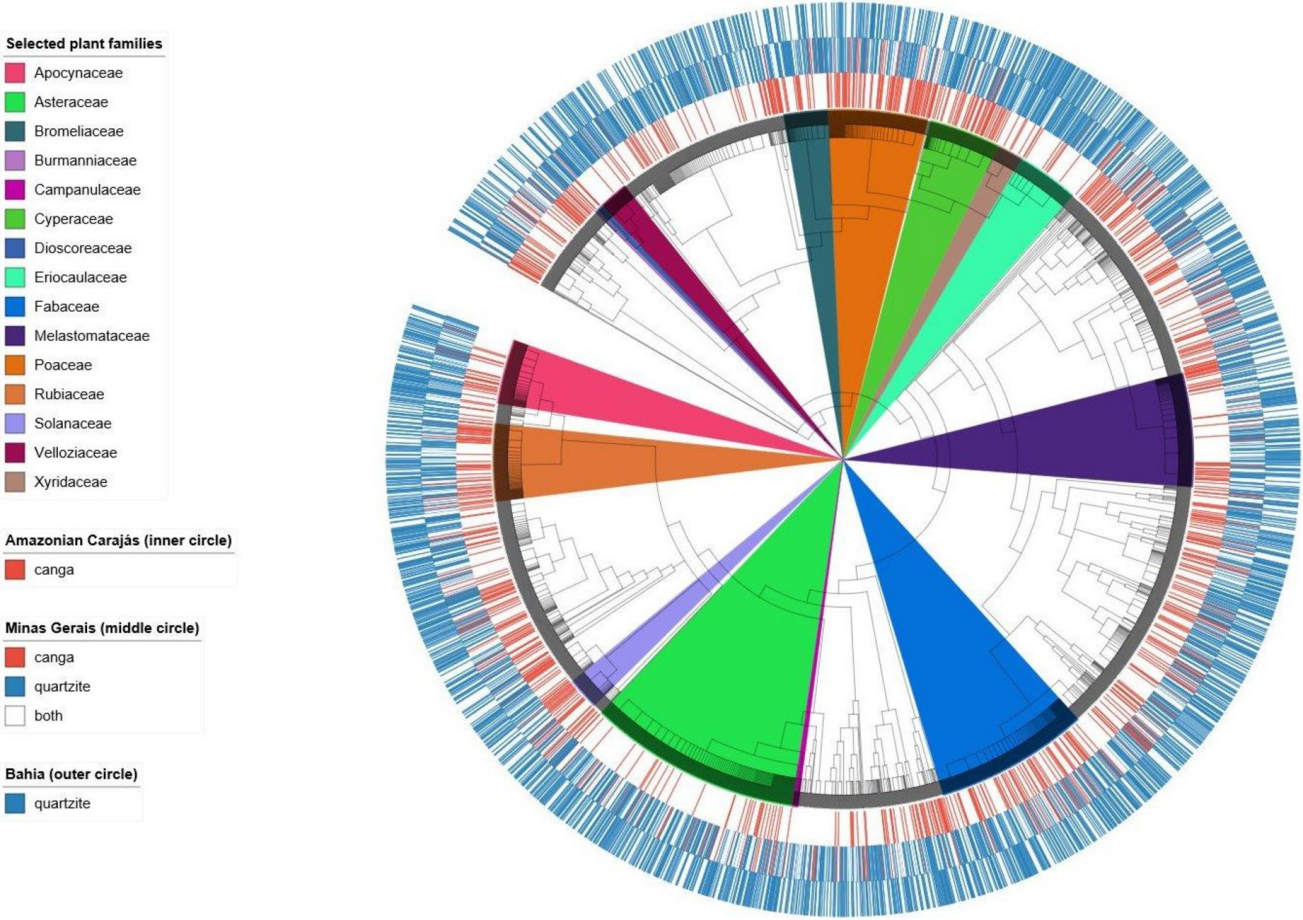
The *campo rupestre* megatree indicating substrate affinities for 4705 species; The outer ring (blue only) represents quartzite in Bahia, middle ring both quartzite (blue) and *canga* (red) in Minas Gerais and inner ring (red only) the Amazonian *canga*. Plant groups mentioned in the results and discussion are highlighted.

Merged lists for the 28 sites included in our analyses yielded 4705 species representing 1070 genera and 157 families, for a total of 11204 occurrences, with species recorded per site ranging from 98 to 1429 and averaging 400 (see Supporting information—S2 Table). Our initial visualization of the phylogenetic tree and species occurrences (Fig 4) suggested clear associations between certain clades and Amazonian Carajás *canga* with relatively few species shared with *canga* of Minas Gerais in eastern Brazil.

### Biogeography between *campos rupestres* in Amazonian Carajás and Eastern Brazil

**Fig 5:**
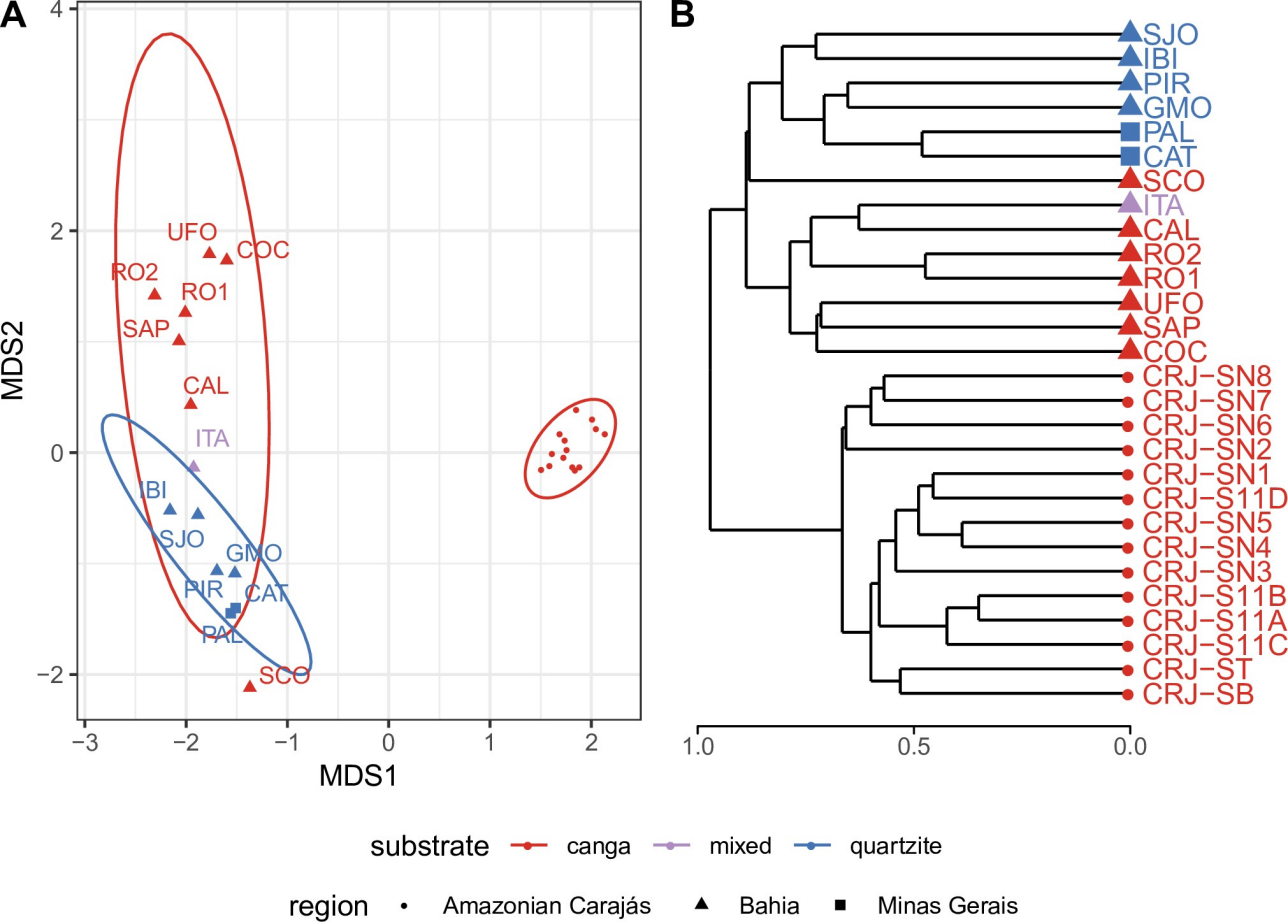
Floristic relationships between the 28 sites analysed including the *canga* of Amazonian Carajás, *canga* of Minas Gerais and quartzite in Minas Gerais and Bahia represented by (a) NMDS using Bray-Curtis distance. Ellipses show 95% confidence limits for delimitation of each group. Final stress for two dimensions: 0.0657927; (b) UPGMA using Bray-Curtis distance showing the floristic relationships between the 28 sites analysed representing the *canga* of Amazonian Carajás, *canga* of Minas Gerais and quartzite of Minas Gerais and Bahia. For abbreviations see Fig 1 caption.

The broadscale biogeographical comparison showed that the *canga* flora of Amazonian Carajás formed a cohesive group, clearly distinct from the flora of Eastern Brazil, whether on *canga* or quartzite. The flora from Amazonian Carajás sites were clearly separated from all other sites on the first axis of the NMDS plot (Fig 5A) and in the deepest branch of the UPGMA (Fig 5B) grouping. Within Minas Gerais, the second axis of the NMDS plot separated *canga* from quartzite, with one notable exception, Serra do Condado (SCO). Thus, *canga* sites in Amazonian Carajás harboured a very different set of species from those occurring on *canga* in Minas Gerais, with *canga* in Minas Gerais being more similar to quartzite in Minas Gerais and Bahia than to *canga* in the Amazon.

ANOSIM showed significant (p = 0.001) values for distinctness of the regional groups formed (Amazonian Carajás *versus* Minas Gerais *versus* Bahia) as well as for substrate (*canga versus* quartzite) (p = 0.01).

### Differential representation of lineages across areas

**Fig 6:**
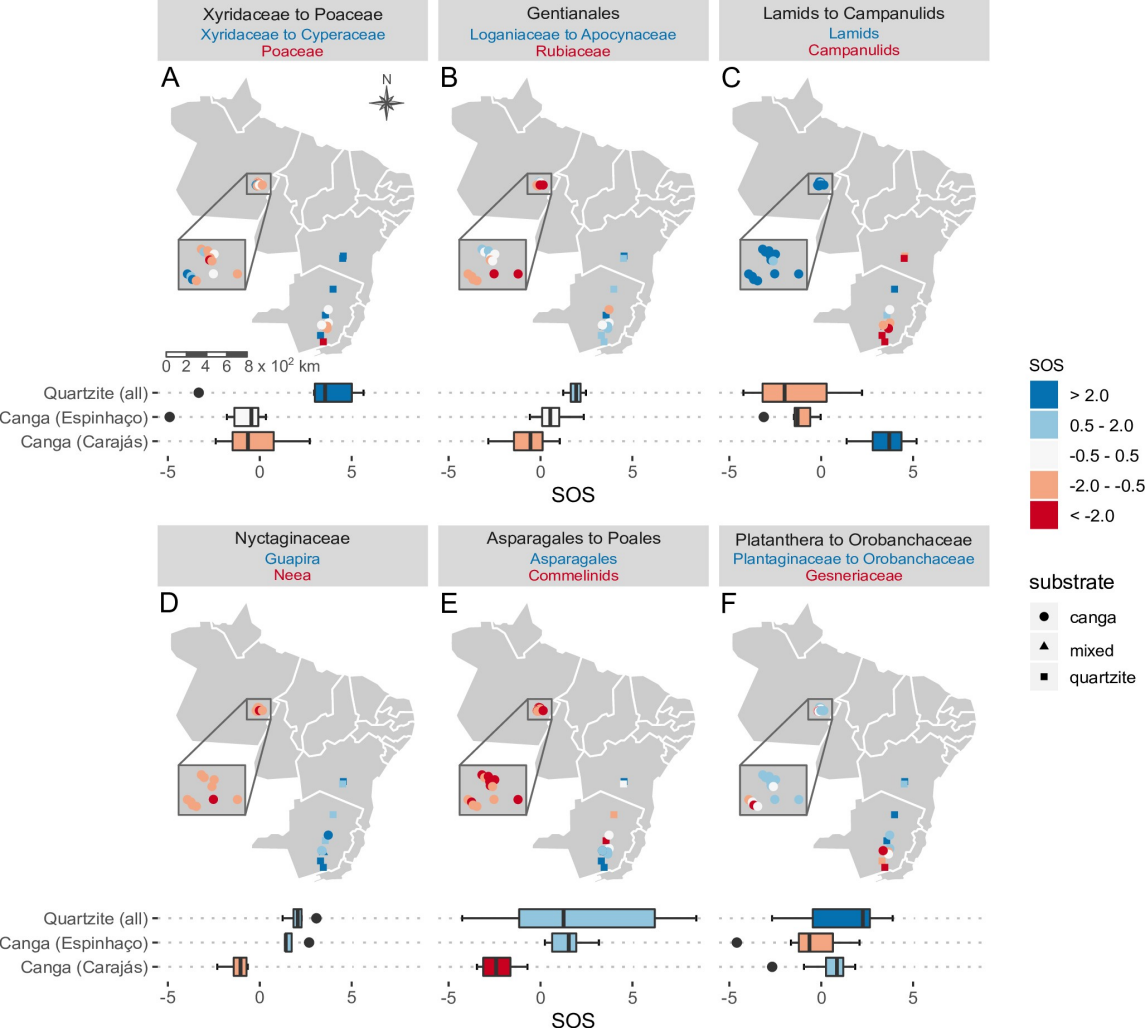
Six examples of differential representations showing contrasting over-represented clades in the quartzitic, canga or mixed areas. (a) Xyridaceae to Cyperaceae in quartzite versus Poaceae in canga; (b) Loganiaceae to Apocynaceae in quartzite versus Rubiaceae in canga; (c) Lamids in the Amazon × Campanulids in Eastern Brazil; (d) Family Nyctaginaceae, genus *Guapira* in the Amazon × *Neea* in Eastern Brazil; (e) Asparagales in the Atlantic Rainforest × Commelinids elsewhere; (f) Plantaginaceae to Orobanchaceae in campo rupestre × Gesneriaceae in the Atlantic Raiforest. SOS = specific overrepresentation score.

The NODIV analysis found 20 pairs of sister groups that differed significantly in distribution across our study sites. A general pattern was observed regarding substrate, with certain major clades across the phylogenetic tree being better represented on quartzite while others were better represented on *canga*. Specifically, in Monocots, the Xyridaceae-Eriocaulaceae clade was better represented on quartzite while Poaceae and Cyperaceae were better represented on *canga* both in the Amazon and in Minas Gerais (Fig 6A). Among Eudicots, the Loganiaceae-Apocynaceae clade was better represented on quartzite while the Rubiaceae were more strongly represented on *canga*, especially in Amazonian Carajás (Fig 6B).

A few clades showed significant differential representation between Amazonian Carajás and Eastern Brazil. A notable example was the Lamid clade, strongly represented in the Amazonian *canga* sites, while the sister clade, the Campanulids, tended to be better represented in Eastern Brazil (Fig 6C). At a finer taxonomic scale, within the Nyctaginaceae, all but one occurrences of the genus *Neea* were in Amazonian *canga* while *Guapira* was reported only for Eastern Brazilian sites (Fig 6D).

A third pattern was evident in certain plant groups found to be better represented in southern Minas Gerais, such as Orchidaceae (Fig 6E), Bromeliaceae, Gesneriaceae and Ericaceae (Fig 6F), while their sister clades Commelinids, Rapataeaceae to Poaceae (Fig 6E), and Plantaginaceae to Orobanchaceae (Figs 3F and 3G and 6F), and Symplocaceae were over-represented elsewhere in Brazil.

### Dissecting biogeographical patterns in Carajás

**Fig 7:**
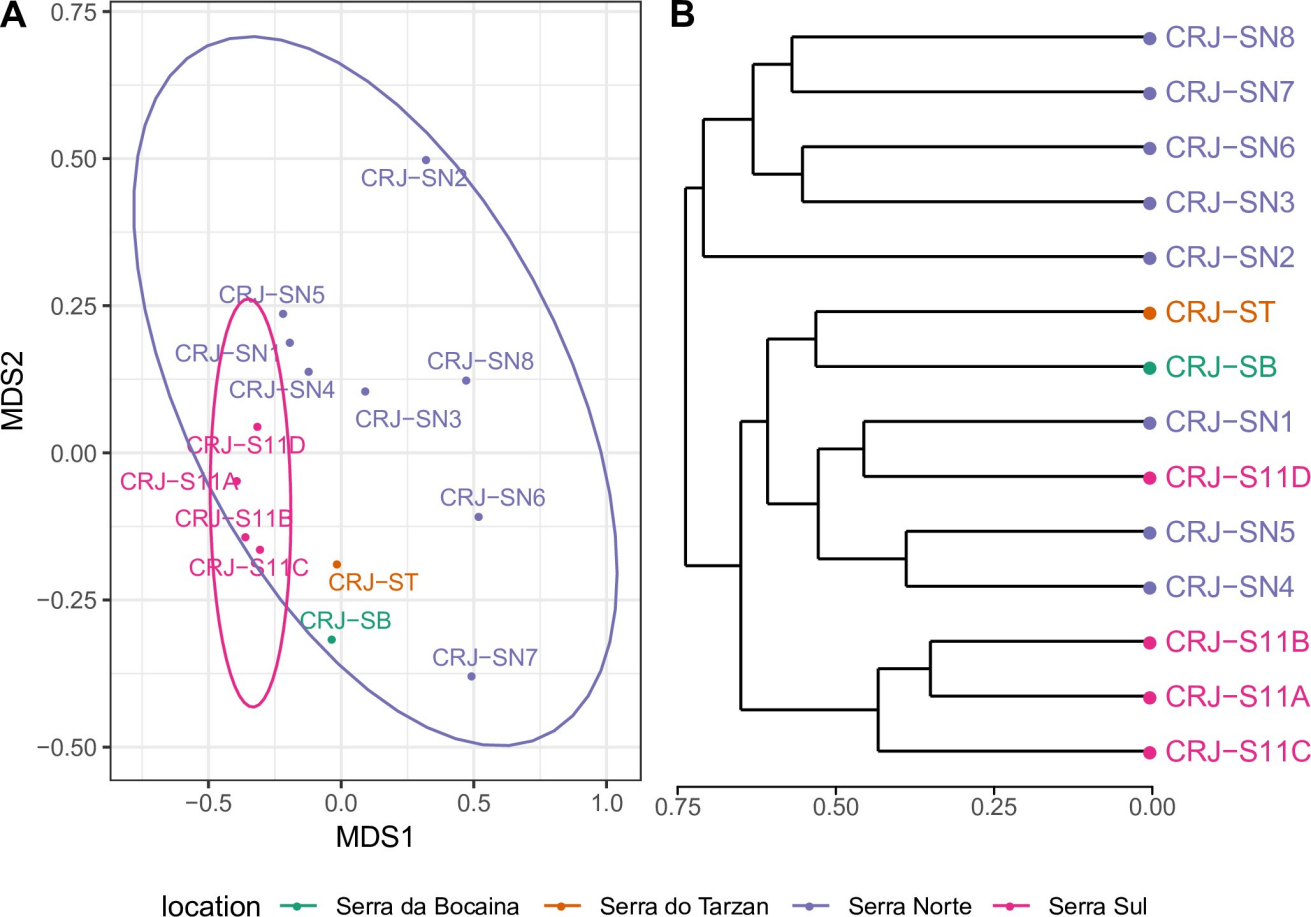
(a) NMDS ordination using Bray-Curtis distance for the individual mountaintops in Amazonian Carajás. Total stress = 0.1439546. Ellipses show 95% confidence limits for delimitation of each group; (b) UPGMA grouping using Bray-Curtis distance of individual mountaintops in Amazonian Carajás. For abbreviations see caption of Fig 1.

Our analysis showed clear differences in floristic composition between northern and southern mountaintops in Amazonian Carajás. The flora of the Serra Sul was relatively homogeneous, with sites very little differentiated on the first NMDS axis and only slightly more differentiated on the second axis (Fig 7). In contrast, the flora of the Serra Norte showed greater floristic dissimilarity between sites (Fig 7A). While five of the eight Serra Norte sites formed a well defined group (Fig 7B), the three remaining Serra Norte sites grouped with the Serra Sul, Serra da Bocaina and Serra do Tarzan.

Rarefaction, extrapolation and richness estimates demonstrated that the intensive collection effort that took place before and during the *Flora of Carajás* project [19,22,24] produced a greatly expanded, but not yet complete angiosperm inventory for the *canga* of Carajás. While 830 native species (plus 25 invasive) are now documented from the 14 mountaintops already surveyed, the total richness estimated varies from 958 species (Chao 2) to 1085 species (Jacknife 2). Doubling the sampling effort is predicted to result in a total of 933 species (see Supplementary information–S3 Fig and S3 Table).

Within the Amazonian Carajás *canga*, our *nodiv* analysis revealed differential representation of certain clades between major locations. Among Monocots, Araceae and related families were significantly better represented in the Serra Sul than at other major locations in the Amazonian *canga*, which had a more marked presence of taxa from Pandanales to Poales clade (Poaceae, Eriocaulaceae, Cyperaceae; and Orchidaceae). Among early branching Angiosperms, the paleoherbs (Piperaceae, Aristolochiaceae) tended to be better represented in the Serra Sul than in other major locations which had better representation of woody families, such as Annonaceae and Lauraceae. Similarly, the Poaceae over-representation compared to Xyridaceae, Cyperaceae and Eriocaulaceae observed for Serra Norte, Serra da Bocaina and Serra do Tarzan was less clear in the Serra Sul outcrops (S2 Fig).

## Discussion

**Fig 8:**
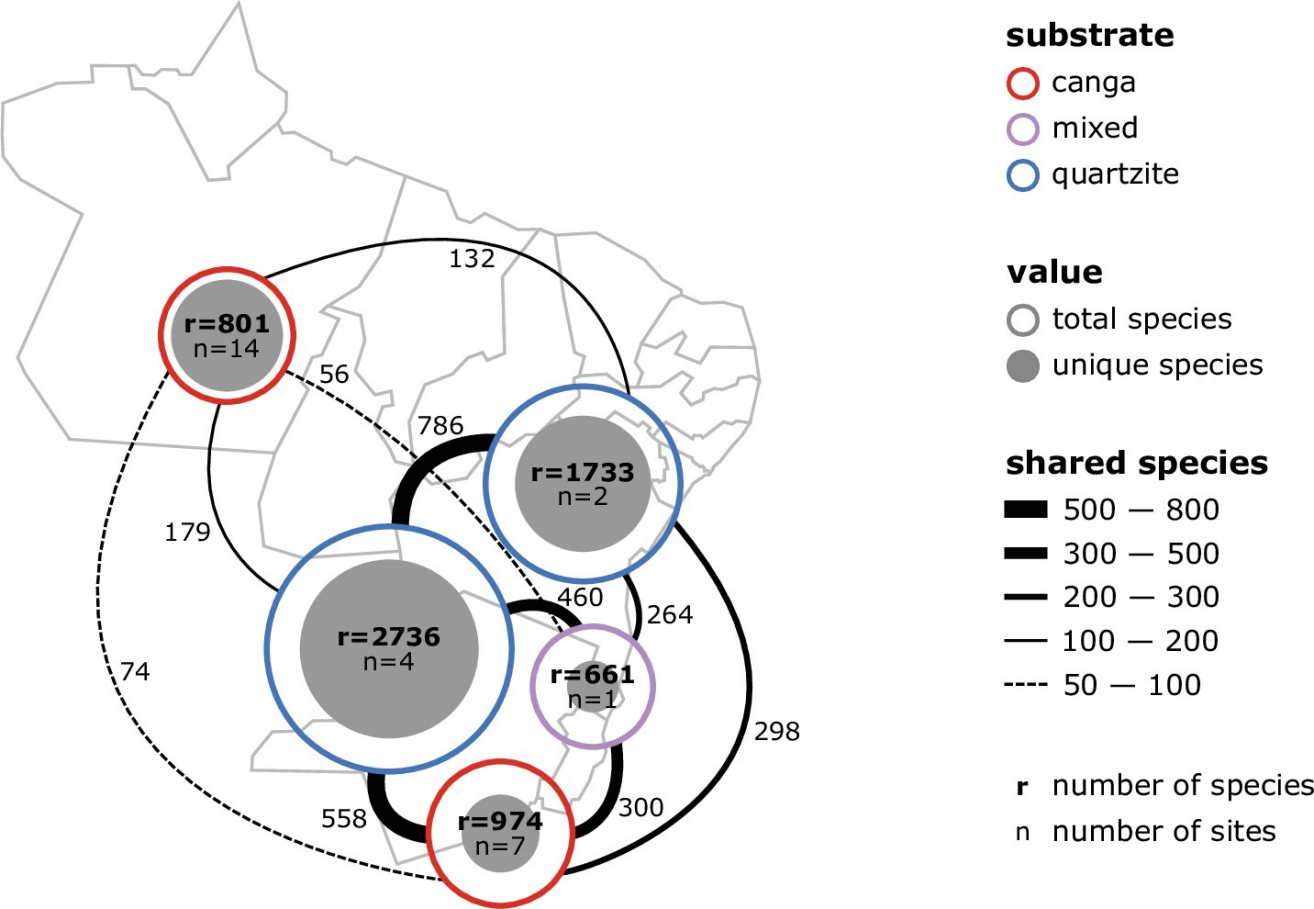
Diagram of sites grouped during the analysis, showing total species richness for merged groups of sites (r), number of combined sites (n) for each group and relative numbers of species shared between groups. From top right: Amazonian *canga* (red), Bahia (blue), Minas Gerais quartzite (blue), Minas Gerais, *canga* and quartzite (lilac) and Minas Gerais *canga* (red).

Using a combination of floristic comparisons by multivariate analysis and phylogenetic tree reconstruction we showed that the flora of *campos rupestres* in Brazil is extremely heterogeneous. While the *campos rupestres* on *canga* and quartzite of Eastern Brazil have long been considered very rich and diverse, we revealed here for the first time that floristic dissimilarity was even higher between Eastern Brazil and Amazonian Carajás than within the communities already recognized as hyperdiverse in Eastern Brazil (Fig 8). This has profound consequences for conservation planning of these localities: conserving small portions of these highly threatened regions is not an effective way to safeguard *campo rupestre* biodiversity.

Our study is a first step towards recognizing the heterogeneity between individual mountaintops in Amazonian Carajás *canga* and may be used as a guide towards a more comprehensive conservation strategy. For example, the heterogeneity we found in Amazonian Carajás *canga* suggests that, although important, the newly established national park (PNCF), that includes only two canga outcrops (SB and ST) may not be sufficient to conserve regional biodiversity because the heterogeneity in Amazonian Carajás is so high.

### Biogeography of *campos rupestres* in Amazonian Carajás and Eastern Brazil

Plant communities on *canga* in Minas Gerais show greater floristic similarity to each other than to most plant assemblages in quartzitic *campo rupestre* in Eastern Brazil [3]. Here, our extended geographical coverage showed that Minas Gerais *canga* and the Amazonian Carajás *canga* did not form a cohesive floristic group. Plant communities on *canga* in Eastern Brazil were more similar to those on quartzite in Eastern Brazil than to *canga* in Amazonian Carajás (see Figs 4 and 5A and 5B).

The over-representation of Xyridaceae (Fig 3A) and Eriocaulaceae (Fig 3B) reported for the *campos rupestres* on quartzite in the Eastern Brazil [3] was corroborated by our study. Use of the *nodiv* package [41] allowed us to visualize and pinpoint nodes at which sister clades which were differentially represented across our sites diverge. For example, it was possible to ascertain that within Monocots, Xyridaceae and Eriocaulaceae were over-represented in quartzitic *campo rupestre*, while Cyperaceae and Poaceae (Fig 3C) were better represented in *canga* both in Amazonian Carajás and in Minas Gerais. Similarly, within Eudicots, the “Apocynaceae to Loganiaceae (Fig 3D)” clade (Gentianales) was over-represented on quartzite, while its sister clade Rubiaceae (Fig 3E) was over-represented in *canga* sites. These patterns present a sharp contrast to our floristic results which showed strong dissimilarity between the Amazonian Carajás and Minas Gerais *cangas*. Thus, the phylogenetic perspective complemented the floristic analysis by revealing patterns originating from deeper nodes reflecting associations between certain lineages and substrates. Identifying the processes underlying these patterns is beyond the scope of the current study, but given the ancient nature of these landscapes, the great distances between them, the relatively harsh character of the environment for plants, and the present day hyperdiversity, it is likely that dispersal limitation, environmental filtering and in situ speciation have all played a part in shaping current plant communities, consistent with OCBIL theory [16]. Floristic studies in banded ironstone inselbergs in Western Australia [45] have revealed comparable high richness and endemism influenced by soil chemistry, as well as spatial and climatic gradients.

Notwithstanding the lineage-substrate associations discussed above, our phylogenetic analyses also showed marked geographic pattern in the distribution of some clades, as exemplified by significant differential representation between *campo rupestre* in Amazonian Carajás and Eastern Brazil. For example, it is possible that certain traits found in the Asteraceae (e.g., presence of fire-resistant xylopodia) might contribute to high diversity of this group in Eastern Brazil, independent of substrate. Although very species-rich in most Brazilian biomes, Asteraceae has much lower species diversity in the Amazon [30].

Surrounding biome may strongly influence the representation of lineages in certain sites, as seen in the over-representation of primarily epiphytic plant families such as Orchidaceae, Bromeliaceae and Gesneriaceae (Fig 3H) in our southern sites located within the Atlantic Forest, corroborating Freitas et al [46].

### Floristics and endemism in the Amazonian Carajás *canga*

Despite the striking physiognomic similarities between different *campos rupestres* and analogous adaptations found throughout geographically distant *campo rupestre* floras [11], we found that the floristic composition of this vegetation on Amazonian Carajás *canga* was extremely dissimilar to that found in *campo rupestre* in Eastern Brazil, both on *canga* and quartzite. These results are consistent with the very limited overlap between seed plant species from the *canga* of Carajás, and those from the *canga* in Corumbá (17 species of the 174 reported by [47] or the Iron Quadrangle [48,49] with only 13 widespread species common to all regions [19].

Further analysis of Amazonian Carajás *canga* sites revealed even more heterogeneity, with the four Serra Sul sites showing strong floristic similarity while the eight Serra Norte sites showed greater floristic heterogeneity, reflecting the physical separation between the Serra Norte outcrops in contrast to the more continuous terrain of the Serra Sul (Fig 1). The Serra da Bocaina and Tarzan sites, the only ones encompassed in a fully protected area, were floristically most similar to each other and then to the Serra Sul sites. Since open-cast mining is underway both in Serra Norte (N4, N5) and Serra Sul (S11D), and expansion of mining is under active consideration, it is important to note that *canga* floras of Serra Norte and Serra Sul clusters also ought to be protected if the full range of biodiversity of the Amazonian Carajás *canga* is to be conserved.

Endemism in the Carajás region, first detected in 1969 during the first botanical survey in the then recently discovered mineral province, resulted in the description of *Parapiqueria cavalcantei* [50], *Cavalcantia glomerata* [51], *Perama carajasensis* [52] and *Ipomoea cavalcantei* [53]. More than 60 taxa have been described based on specimens collected in the Carajás region over the last 50 years (Giulietti et al. under review) and discovery of species new to science continues to the present day, e.g. *Anemopaegma carajasensis* [54], *Peperomia albopilosa*, *P*. *pseudoserratirhachis* [55]. In spite of ongoing discovery of new species, our rarefaction and extrapolation analyses based on 14 sites suggested that current knowledge is reasonably robust, as the species accumulation curve started to stabilise at 11–12 sites and doubling the sampling effort to a total of 28 sites predicted an increase in known species richness to 933 species, when conservatively estimated. This suggests that the *Flora of Carajás* project [19,22,24] has been successful in representing the region´s seed plant diversity. Although the projected increase in species numbers may appear modest (c. 103 species, representing a 12% increase to the current total of 830), species yet to be discovered on these mountaintops are likely to be range-restricted, potentially endemic to Carajás and probably of high conservation concern [56].

### Anomalous sites and sampling effects

Our analysis showed that dissimilarity between *canga* sites in Minas Gerais and quartzite sites in Minas Gerais and Bahia were relatively small when compared to dissimilarity between *canga* sites in Amazonian Carajás and Eastern Brazil. Although these overall patterns were clear, certain notable exceptions are of interest, not least because they suggest opportunities for further work. The most striking exception emerging from our analysis was the Serra do Condado site, which differs from other Minas Gerais *canga* sites, showing greater similarity to quartzitic *campo rupestre*. The geographic position of this site, well outside the geological boundaries of the Iron Quadrangle, may explain the dissimilarities found, however sampling effects could also explain these divergent results. The differential representation analysis helped to illuminate the position of this site, which showed over-representation of groups rich in woody clades such as Superrosids, Magnoliids, Lauraceae and Annonaceae, Symplocaceae, and also of the Araceae, a family rich in hemi-epiphytes found in forest. This may reflect the particular focus the list of the Serra do Condado survey [57] on woody species. Sampling effects may also underlie the apparent over-representation of the carnivorous plant family Lentibulariaceae at Pico das Almas (nodiv n. 5), as year-round fieldwork at this site in the late 1980s was directed towards a global monograph of *Utricularia* [58].

Not all mountaintops in the Carajás region have been sampled with equal intensity and the sites (S11D, N4, N5, N1) where fieldwork was more concentrated, as they were located in more accessible areas, provided a particularly important contribution to the current portrait of the area’s diversity. Both Serra do Tarzan and Serra da Bocaina are less accessible than Serra Norte and Serra Sul and were possibly under-collected.

Environmental heterogeneity and specialisation, in the case of this study represented by impacts of *canga* substrates on local biodiversity, play an important role as drivers of biodiversity. Understanding of such heterogeneity is needed to inform conservation strategy. In our investigation of *canga* across different geographic regions in Brazil, we have underscored the fact that the combination of specialised substrate and broader patterns of biogeography result in considerable heterogeneity in biodiversity across *canga* sites in different parts of Brazil. Thus, conservation planning must take into account the pressing need for provision of protected area coverage for *campos rupestres* sites both in Amazonian Carajás and Eastern Brazil [59]. In the latter, it is necessary to distribute the reserves on quartzite and *canga* substrates in order to encompass not only species, but also lineages. The urgent need for reserves for Amazonian Carajás *canga* was recognized and addressed, at least in part, by the recent creation of the new national park (PNCF). However, current sampling does not provide sufficient reassurance that these *canga* areas now benefitting from full legal protection adequately represent the regional *canga* flora.

## Supporting information

S1 Fig Methodology flowchart. (PDF)

S2 Fig Additional differential representation figures comparing floristic groups within the areas. (PNG)

S3 Fig Rarefaction graph for Carajás showing extrapolation and estimated figures. (PDF)

S4 Fig Phylogenetic tree obtained for Carajás showing and Espinhaço flora in PDF format. (PDF)

S1 Table Data matrix for Carajás. (CSV)

S2 Table Combined data matrix including Carajás and Espinhaço flora. (CSV)

S3 Table Rarefaction data for Carajás. (TXT)

S1 Text Script used for the analyses. (R)

S2 Text R Phylogenetic tree obtained for Carajás and Espinhaço flora. (TXT)
