# Supplementary figures and images for: Plotting a future for Amazonian canga vegetation in a campo rupestre context
Source: PLoS One. 2019 Aug 5;14(8):e0219753. doi: 10.1371/journal.pone.0219753 (PMC6681939; doi:10.1371/journal.pone.0219753)

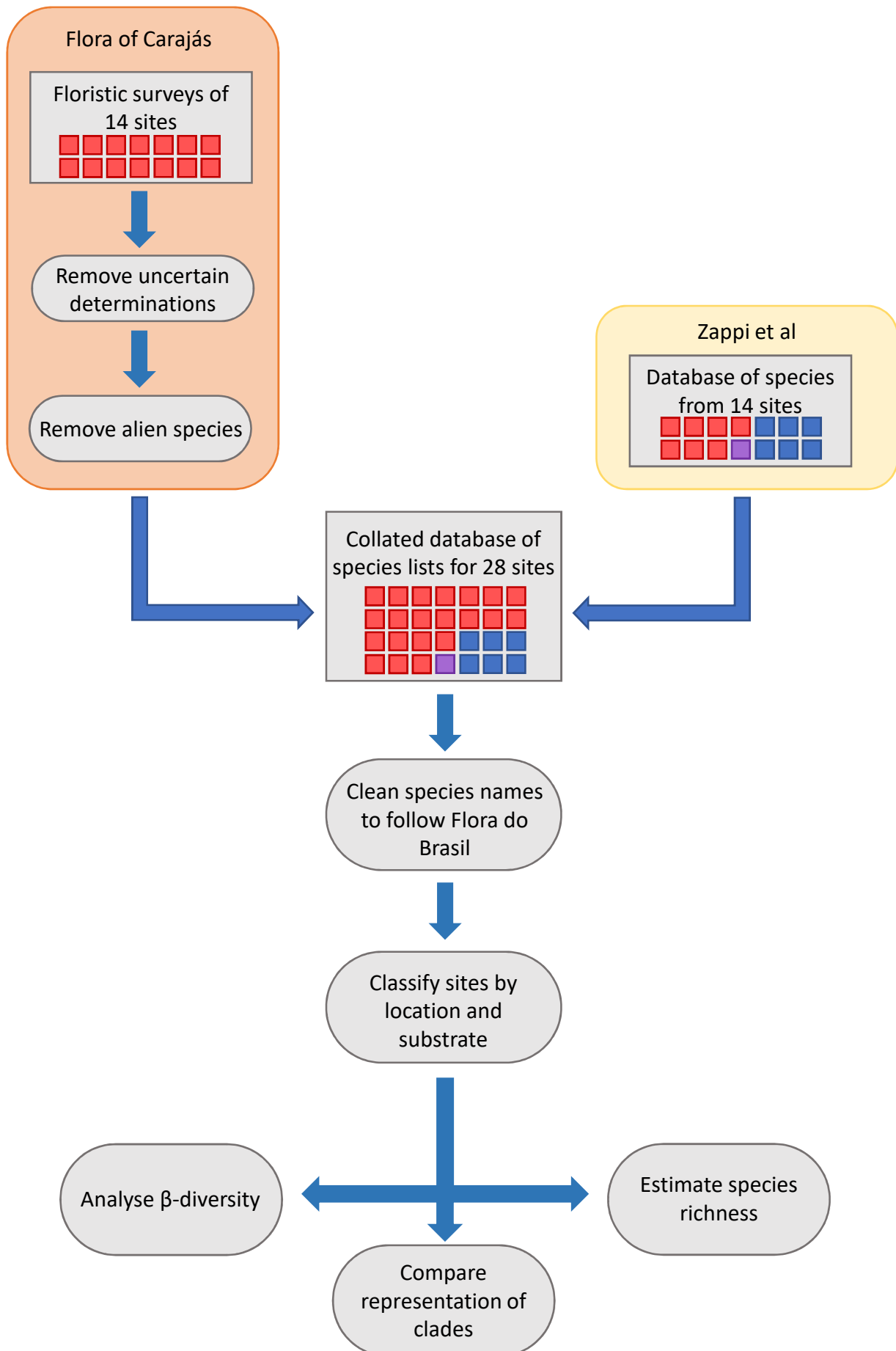

Supplement: S1 Fig — (PDF) [file pone.0219753.s001.pdf]

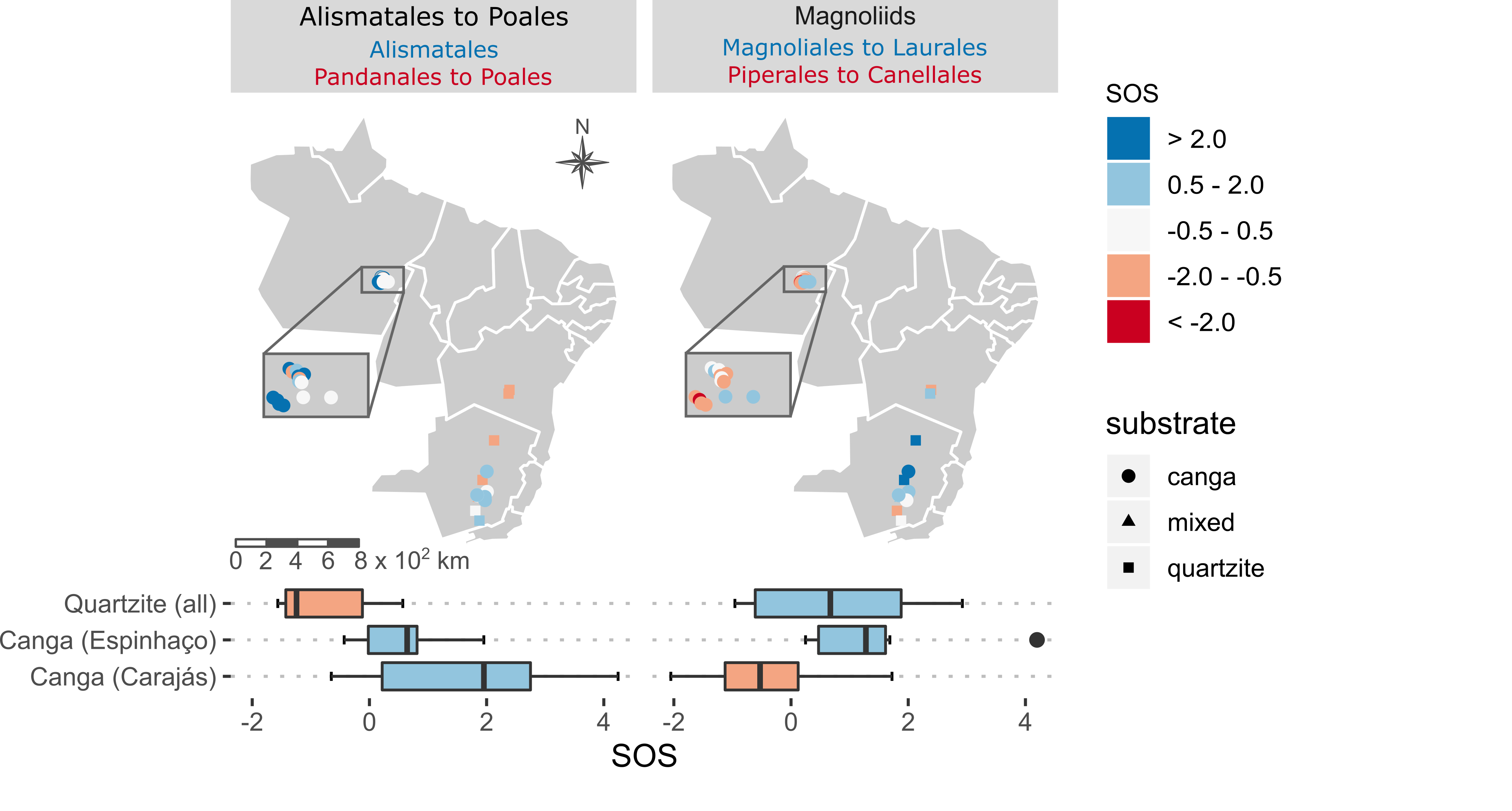

Supplement: S2 Fig — (PNG) [file pone.0219753.s002.png]

Tree scale: 10

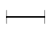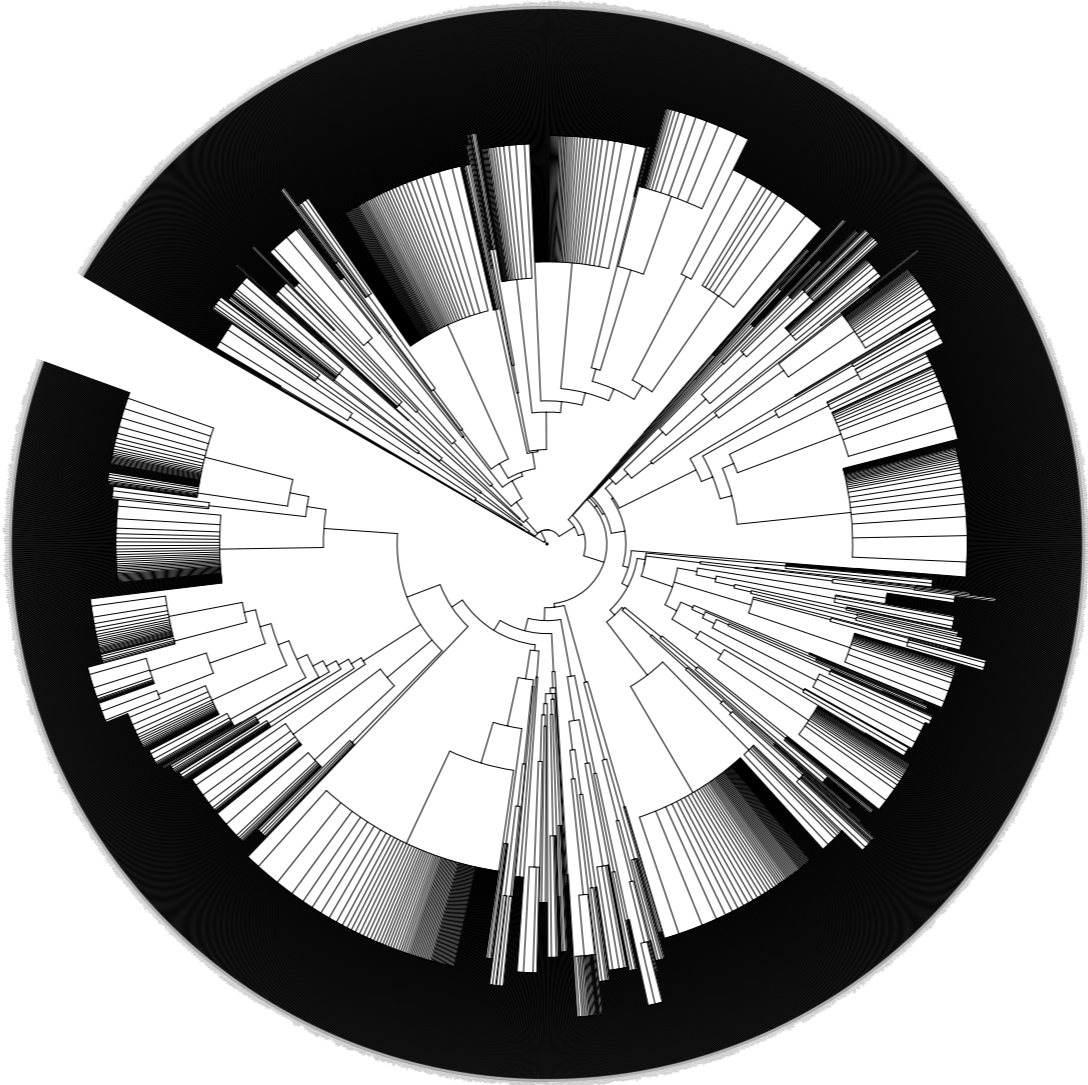

Supplement: S4 Fig — (PDF) [file pone.0219753.s004.pdf]
