# Supplementary material for: Plotting a future for Amazonian canga vegetation in a campo rupestre context
Source: PLoS One. 2019 Aug 5;14(8):e0219753. doi: 10.1371/journal.pone.0219753 (PMC6681939; doi:10.1371/journal.pone.0219753)
Supplement: S3 Fig — (PDF) [file pone.0219753.s003.pdf]

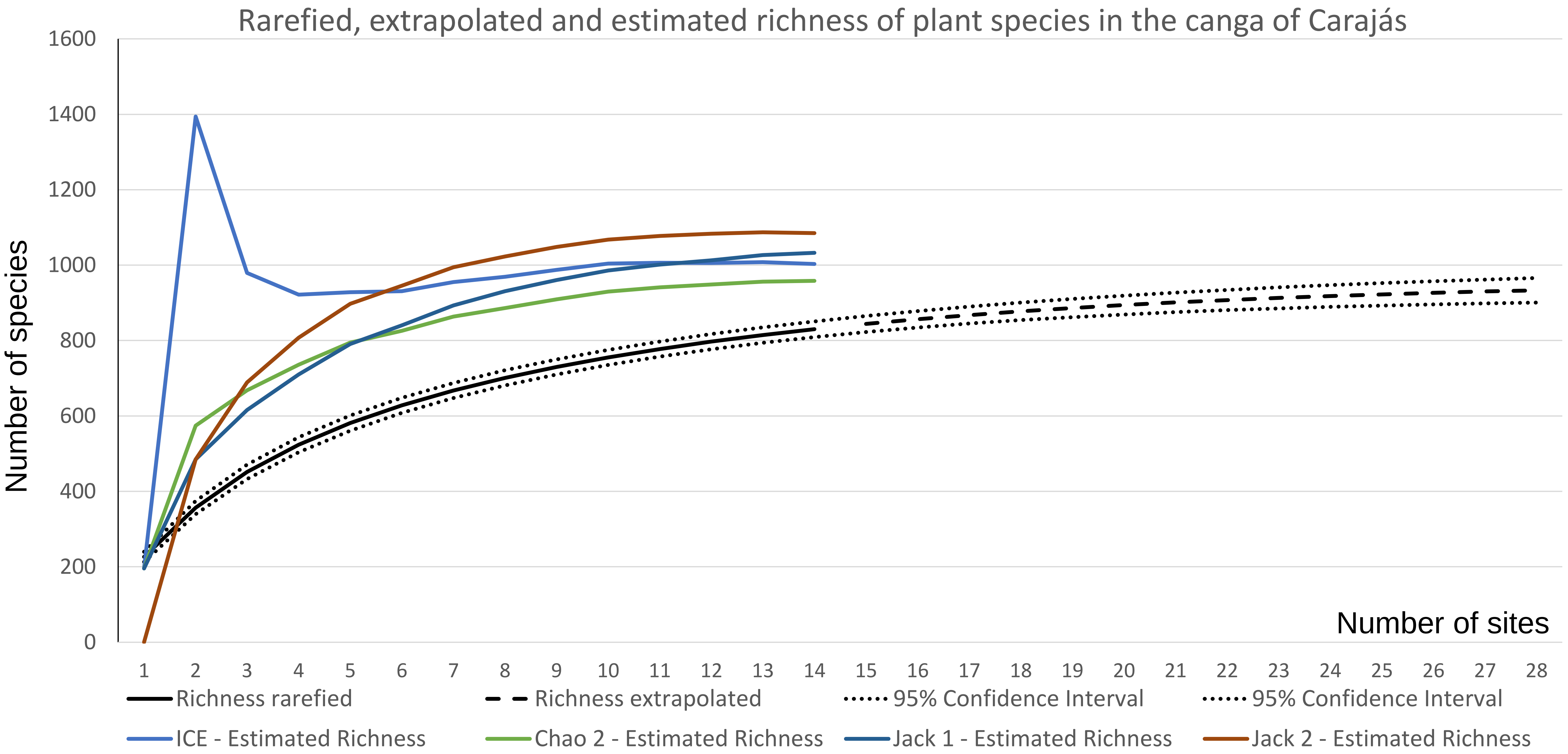

Rarefied (interpolated), extrapolated and estimated richness for flora sampling in individual mountaintops with canga in Carajás. 830 native species were recorded in 14 sites, while a total of 933 species are expected if sampling is extended to include a total of 28 sites. Total asymptotic estimated richness varies from 958 to 1085 species.
